# Impact of Different Pneumoperitoneum Pressures on Internal Carotid Artery Blood Flow and Cerebral Oxygenation in Laparoscopic Sleeve Gastrectomy

**DOI:** 10.1007/s11695-026-08736-9

**Published:** 2026-05-22

**Authors:** Furkan Doğan, Fatma Celik

**Affiliations:** Present Address: Department of Anaesthesiology and Reanimation, Faculty of Medicine, Firat University, Elâzig, Turkey

**Keywords:** Severe obesity, Bariatric surgery, Carotid artery, Cerebral hemodynamics, Cerebral oxygenation, Near-infrared spectroscopy

## Abstract

**Background:**

High intra-abdominal pressure (IAP) during bariatric surgery may compromise cerebral perfusion. This study evaluates the impact of 12 vs. 16 mmHg pneumoperitoneum on internal carotid artery (ICA) hemodynamics and cerebral oxygenation (rSO_2_) in patients with severe obesity undergoing laparoscopic sleeve gastrectomy (LSG).

**Methods:**

In this prospective, randomized, double-blind study, 67 patients undergoing LSG were allocated into two groups: Group 12 (12 mmHg, *n* = 34) and Group 16 (16 mmHg, *n* = 33). Internal carotid artery velocity-time integral (VTI), internal carotid artery blood flow (ICABF), and rSO_2_ were recorded at seven time points (T_0_ to T_6_). Acute positional and cumulative pressure effects were analyzed using delta (Δ) change values.

**Results:**

Systemic hemodynamics remained stable across groups (*P* > 0.05). However, Group 16 showed significantly greater cumulative reductions in both ICA-VTI and ICABF compared to Group 12, particularly during the T_0_–T_6_ period (*P* < 0.001). Specifically, the decline in ICABF for Group 16 was significantly more pronounced at T_0_–T_6_ (-161.89 vs. -107.41; *P* = 0.004) and T_2_–T_4_ (-87.95 vs. -72.06; *P* = 0.036). The combined effect of pneumoperitoneum and reverse Trendelenburg position (T_2_–T_3_) induced a significantly greater acute reduction in rSO_2_ in Group 16 (Δ: -1.82) than in Group 12 (Δ: -0.24; *P* = 0.029).

**Conclusion:**

Elevated 16 mmHg IAP exerts a subclinical hemodynamic burden by straining cerebral reserves through cumulative ICABF reduction and acute rSO_2_ declines. An initial pressure of 12 mmHg may be recommended to optimize neuro-hemodynamic stability, with subsequent adjustments tailored to surgical requirements and patient needs.

**Graphical Abstract:**

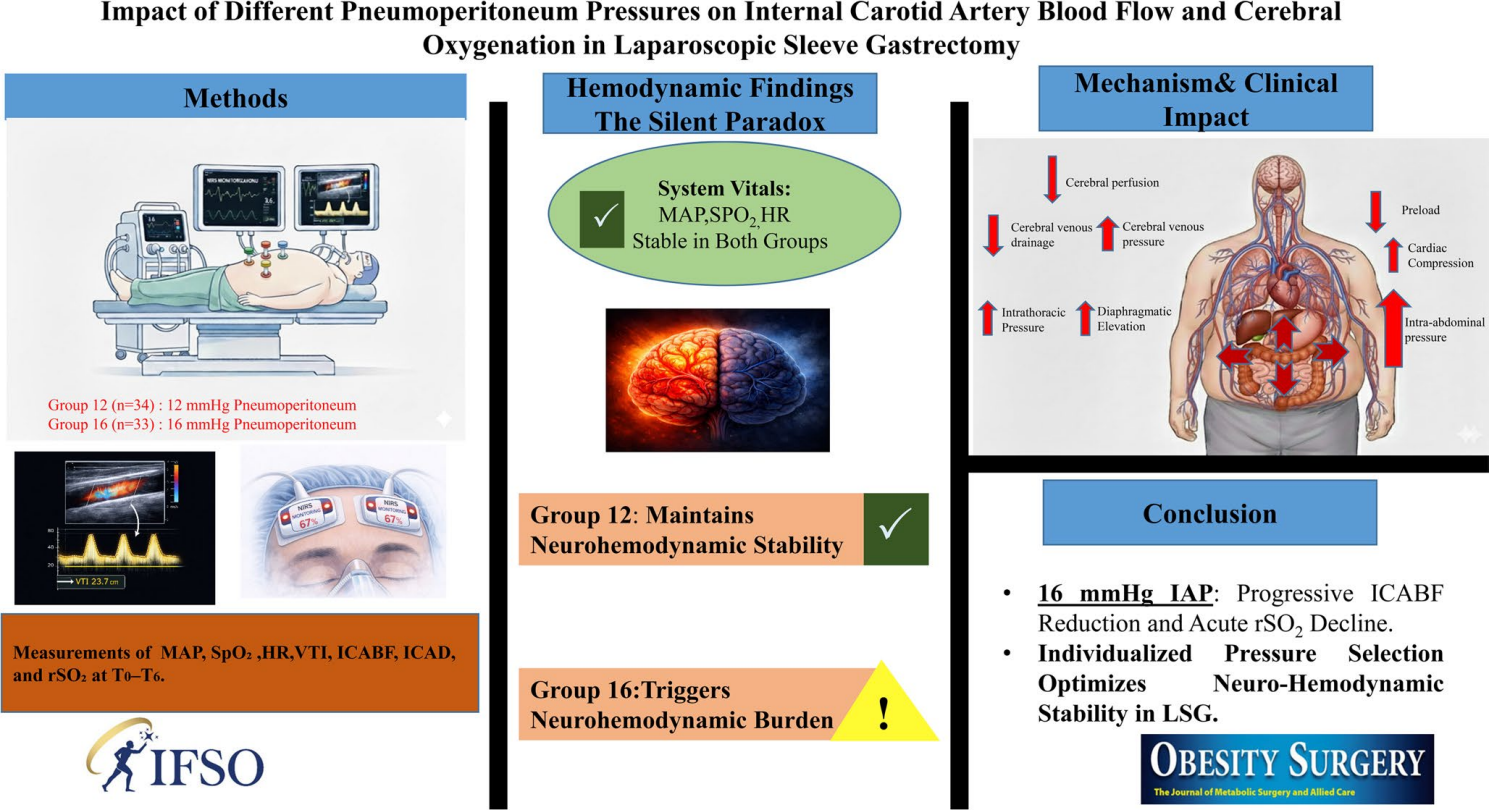

## Key Points

Among patients with severe obesity, elevated intra-abdominal pressure at 16 mmHg results in a cumulative reduction in internal carotid blood flow (T0–T6), despite stable systemic hemodynamic parameters.

The combination of high-pressure pneumoperitoneum and reverse Trendelenburg position is associated with significantly more pronounced declines in cerebral oxygenation ( Δ rSO2).

Provided that an adequate surgical field is maintained, the lowest effective intra-abdominal pressure (preferably 12 mmHg) should be prioritized to preserve cerebral perfusion reserves in individuals with severe obesity.

## Introduction

Laparoscopic sleeve gastrectomy (LSG) is a leading global surgical intervention for managing severe obesity [1]. This procedure typically requires combining CO_2_ pneumoperitoneum with the reverse Trendelenburg (RT) position [2]. However, in severe obesity, this positioning can impose a substantial hemodynamic burden. The combined effects of RT and abdominal insufflation may obstruct cerebral venous drainage and alter perfusion pressure [3, 4].

Internal carotid artery (ICA) Doppler assessment and near-infrared spectroscopy (NIRS) are reliable, non-invasive methods for monitoring these subclinical changes. Although a 15 mmHg intra-abdominal pressure (IAP) is generally used during LSG, hemodynamic responses may vary significantly due to associated medical problems and cardiovascular alterations linked to severe obesity. To the best of our knowledge, evidence remains limited, as no previous study has specifically compared how 12 mmHg and 16 mmHg IAP levels affect internal carotid artery blood flow (ICABF) and cerebral oxygenation (rSO_2_) in this patient group. While maintaining adequate surgical visibility is a key requirement, tailoring IAP to individual responses may offer a safer surgical approach. This strategy aims to enhance patient safety while preserving an effective operative field [5].

This investigation principally focuses on evaluating the simultaneous effects of 12 and 16 mmHg pneumoperitoneum on ICABF and rSO_2_ during LSG in patients with severe obesity.

The secondary objective is to compare these specific pressures to provide physiological evidence for individualized pressure management. Through this evaluation, we strive to clarify the optimal balance between surgical visibility and neuro-hemodynamic stability.

## Materials and Methods

### Design of the Study and Ethical Considerations

Following approval from the Firat University Faculty of Medicine Local Ethics Committee (Date: 02.10.2024, No: 538680), we initiated this prospective, randomized, double-blind clinical trial. The study was conducted at Firat University Faculty of Medicine Hospital between May 1, 2025, and September 1, 2025, in strict adherence to the Declaration of Helsinki. Each participant signed a written informed consent form after a comprehensive briefing on the study protocol. This research was conducted under the auspices of the Firat University Scientific Research Projects Coordination Unit (Project No: TF.23.02).

## Patient Selection

A total of 77 patients with severe obesity scheduled for LSG were enrolled in the study.

Inclusion criteria: (1) Elective primary LSG surgery; (2) ASA physical status grade III; (3) age ≥ 18 years; and (4) body mass index (BMI) ≥ 40 kg/m^2^.

Exclusion criteria: (1) prior bariatric surgery; (2) concomitant surgical procedures; (3) neck deformities limiting ultrasound access; (4) carotid artery plaque or stenosis; (5) history of ischemic stroke; (6) multiple previous abdominal surgeries; (7) ejection fraction < 30%; (8) severe valvular disease; (9) advanced renal or hepatic failure; (10) skin pigmentation obstructing NIRS signals; (11) requirement for a tidal volume greater than 8 mL/kg based on ideal body weight (IBW); and (12) anticipated difficult airway.

An a priori power analysis using G*Power software (v3.1.9.2) determined the minimum sample size required for this study. Following previous literature on carotid hemodynamics, a power analysis (α = 0.05, 1-β = 0.80) indicated a minimum of 32 patients per group (*n* = 64).

**Fig. 1:**
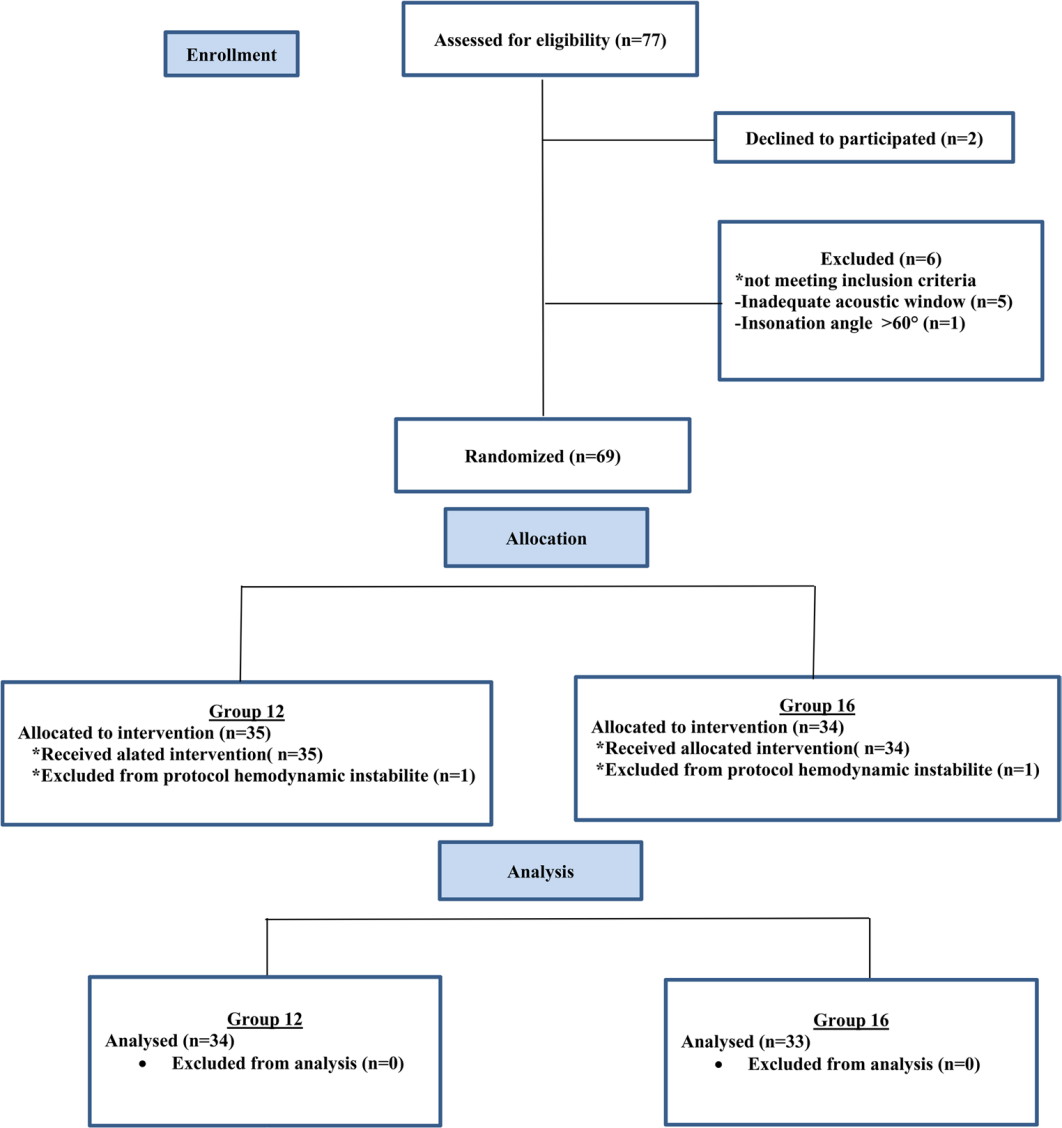
Flow diagram

We performed random group allocation using sequentially numbered, opaque, sealed envelopes to ensure unbiased distribution into Group 12 (*n* = 34; IAP maintained at 12 mmHg) and Group 16 (*n* = 33; IAP maintained at 16 mmHg). The study flow, including initial screening of 77 patients and subsequent exclusions, followed CONSORT guidelines (Fig. 1).

## Anesthesia Management and Monitoring

Upon entry to the operating suite, no premedication was administered. All patients underwent standard monitoring, including electrocardiography, SpO_2_, and invasive arterial pressure measurements. For continuous assessment of cerebral oxygenation (cerebral regional oxygen saturation - rSO_2_), NIRS sensors (Masimo Corp. Root^®^ with O3^®^ Regional Oximetry, Irvine, CA, USA) were applied to the bilateral frontal areas to record baseline values. Intra-abdominal pressure was maintained throughout the procedure using a high-flow insufflator (UHI-4, Olympus Medical Systems, Tokyo, Japan).

After three minutes of preoxygenation (100% O_2_, 4 L/min) in the ramped position, we initiated anesthesia. Induction consisted of propofol 2 mg kg, remifentanil 1 µg/kg, and rocuronium 1 mg/ kg, with all doses calculated based on adjusted body weight for severe obesity. Neuromuscular blockade was managed through a standardized rocuronium protocol starting with the initial dose at induction, followed by maintenance doses every 30 min to ensure sustained deep clinical paralysis. Surgical conditions were monitored using patient-ventilator synchrony and continuous surgeon feedback to maintain optimal abdominal relaxation throughout the procedure. Maintenance was achieved using sevoflurane (0.9–1.2 minimum alveolar concentration) in a 50% O_2_-air mixture. Pulmonary ventilation used volume-controlled mode with a tidal volume of 8 mL/kg (IBW) and 8 cmH_2_O positive end expiratory pressure (PEEP). We adjusted the respiratory rate to target an end-tidal carbon dioxide (EtCO_2_of 35–45 mmHg. All surgical procedures were conducted by the same surgical team in a standardized 30-degree reverse Trendelenburg position to maintain consistent surgical exposure and hemodynamic stability.

## Intraoperative Fluid Management and Hemodynamic Targets

All patients received a 500 mL bolus of balanced crystalloid solution during induction as an initial loading dose. Maintenance fluid therapy was standardized throughout the procedure at a constant rate of 3–5 mL/kg/h based on IBW. Hemodynamic goals were defined as maintaining a MAP ≥ 65 mmHg and a heart rate (HR) > 50 beats/min.

To avoid sudden volume shifts that could potentially confound internal carotid velocity-time integral (VTI) measurements, rapid fluid boluses were avoided in cases of hypotension (MAP < 65 mmHg). Instead, a single 8 µg bolus of norepinephrine was administered as rescue therapy. To ensure measurement precision and maintain data integrity, patients who remained unstable despite this treatment or required additional supplemental vasopressors or atropine were excluded from the final analysis.

## Assessment of Internal Carotid Artery Hemodynamics

Internal carotid artery hemodynamics were assessed using a high-resolution ultrasound system (LOGIQ e, GE Healthcare, Chicago, IL, USA) equipped with a high-frequency linear transducer (up to 18 MHz). A single experienced researcher performed all ultrasonographic assessments 1–2 cm distal to the carotid bifurcation. To ensure measurement accuracy, the insonation angle was maintained at ≤ 60°, with the sample volume centered within the vessel lumen.

The diameter (d) of the ICA was obtained via B-mode imaging, and the cross-sectional area (A) was derived using the formula A = π x (d/2)^2^. The velocity-time integral, which represents the stroke distance per cardiac cycle, was obtained by manually tracing the pulse-wave Doppler envelope. The following equation determined the ICABF:

$$\mathrm{ICABF}\;(\mathrm{m}\mathrm{L}/\mathrm{m}\mathrm{i}\mathrm{n})=\mathrm{VTI}\;(\mathrm{c}\mathrm{m})\times\mathrm A\;({\mathrm{c}\mathrm{m}}^2)\times\mathrm{Heart}\;\mathrm{Rate}\;(\mathrm{b}\mathrm{e}\mathrm{a}\mathrm{t}\mathrm{s}/\mathrm{m}\mathrm{i}\mathrm{n})$$

To minimize respiratory-induced fluctuations, VTI and diameter were calculated as the average of three consecutive heartbeats at each time point.

### Procedural Measurements and Timing

A double-blind protocol was followed, where both patients and the statistician remained unaware of the specific IAP (12 or 16 mmHg) administered during the procedure. A single experienced researcher, independent of the surgical and anesthesia teams, conducted all ultrasonographic assessments. The researcher was aware of the IAP settings due to the clinical environment. However, potential bias was minimized by strictly adhering to a standardized measurement protocol.

Demographic data (age, sex, and BMI) and operative characteristics (surgical duration and total fluid balance) were recorded. The effects of IAP on three primary parameter groups were evaluated: systemic hemodynamics (HR, MAP, and SpO_2_), ICABF (vessel diameter and VTI), and rSO_2_. To ensure hemodynamic stabilization, all measurements were recorded 5 min after reaching each of the following seven time points:

T_0_: Baseline (supine, spontaneous breathing)

T_1_: Post-intubation (supine, mechanical ventilation)

T_2_: pneumoperitoneum (12 or 16 mmHg, supine)

T_3_: pneumoperitoneum + RT (Initial)

T_4_: pneumoperitoneum + RT (30 min)

T_5_: pneumoperitoneum + RT (60 min)

T_6_: Desufflation (RT position)

To eliminate the confounding effects of surgical maneuvers or pharmacological interventions (e.g., vasopressor administration), all ICA Doppler measurements were conducted at least 5 min after any intervention to ensure physiological stabilization. The Doppler probe was positioned one to two centimeters distal to the carotid bifurcation, with an insonation angle of less than sixty degrees, and the sample volume centered within the vessel lumen.

### Statistical Analysis

Data analysis was conducted using IBM SPSS Statistics (version 29.0; Armonk, NY, USA). To determine the appropriate testing strategy, we verified data normality using the Shapiro-Wilk test and checked variance homogeneity through Levene’s test. Results for normally distributed parameters are reported as mean ± standard deviation (SD), whereas non-normal data are shown as median (IQR: Q1–Q3). Frequencies and percentages are used to describe categorical variables.

Normally distributed parameters (surgical duration, fluid resuscitation, rSO_2_, VTI, and ICABF) were compared using the independent samples t-test. Non-normally distributed variables, such as BMI and MAP, were analyzed via the Mann-Whitney U test. Pearson’s chi-square test was applied to categorical data such as sex.

We calculated Δ change values between consecutive periods to assess hemodynamic shifts across the seven intervals (T_0_–T_6_). Depending on data distribution, either the independent samples t-test or the Mann-Whitney U test was employed to compare these Δ values between the 12 mmHg and 16 mmHg groups. Statistical significance was established at *p* < 0.05 across all evaluations.

## Results

Of the 77 patients initially screened, ten were excluded: two declined participation, five presented an inadequate acoustic window, one had an insonation angle exceeding sixty degrees, and two failed to meet the standardized hemodynamic stability requirements. Consequently, the final analysis was conducted on 67 patients who strictly adhered to the measurement protocols (Fig. 1). Among the included patients, transient hypotension during pneumoperitoneum induction (T_2_) occurred in only two cases (*n* = 1 in each group), which was promptly corrected with a single dose of norepinephrine as per protocol. Carotid Doppler measurements were recorded only after achieving full hemodynamic stabilization (MAP ≥ 65 mmHg).

Age, sex, BMI, Associated medical problems, surgical duration, and fluid resuscitation volumes were similar between Group 12 (*n* = 34) and Group 16 (*n* = 33) (*P* > 0.05, Table 1). No significant variations were observed in absolute or delta values for SpO_2_, HR, and MAP at any time point (*P* > 0.05, Table 2).

**Table 1:** Baseline demographic and clinical characteristics of the study group

Variables	Group 12(n = 34)	Group 16(n = 33)	*p* value
	*n*(%)	*n*(%)	
Female	18(52.9%)	16(48.5%)	▪ 0.715
Male	16(47.1%)	17(51.5%)	
Associated Medical Problems(Yes)	10(29.4%)	13(39.9%)	▪0.397
	**Median(Q1-Q3)**	**Median(Q1-Q3)**	
Age(years)	29.5(24.3 – 42.8)	35(29 – 40)	▴0.173
Height(m)	1.65(1.60 – 1.75)	1.70(1.60 – 1.78)	▴0.336
Body Weight(kg)	121(108 – 130)	123(107 – 135)	▴0.778
BMI(kg/m^2^)	43.5(42.2 – 46)	42.1(41.4 – 43.6)	▴0.052
	**Mean ± SD**	**Mean ± SD**	
Duration of Operation(min)	95.3 ± 8.3	92.1 ± 9.7	● 0.153
Intraoperative Fluid Volume(mL)	1725 ± 257	1712 ± 334	● 0.860

BMI, Body Mass Index; Group 12 (12 mmHg pneumoperitoneum); Group 16 (16 mmHg pneumoperitoneum)

Data are presented as *n* (%), median (Q1–Q3), or mean ± standard deviation (SD). Analyses were performed using the ▪ Chi-square test for categorical variables, ▴ Mann–Whitney U test for non-parametrically distributed data, and ● Student’s t-test for normally distributed continuous variables. A *p*-value < 0.05 was considered statistically significant

**Table 2:** Intraoperative monitoring of systemic hemodynamics and regional cerebral oxygenation: Absolute values and longitudinal changes across different time points

	**A: HR(bpm)**			**B: MAP(mmHg)**			**C: SpO**_**2(**_**%)**			**D: rSO**_**2(**_**%)**		
	**Group 12**	**Group 16**		**Group 12**	**Group16**		**Group 12**	**Group 16**		**Group 12**	**Group 16**	
	**Mean ± SD**	**Mean ± SD**	***p* value**	**Median** **(Q1-Q3)**	**Median** **(Q1-Q3)**	***p* value**	**Median** **(Q1-Q3)**	**Median** **(Q1-Q3)**	***p* value**	**Mean ± SD**	**Mean ± SD**	***p* value**
**T₀**	83 ± 9.5	83 ± 16	^●^0.932	95(84.3–106)	94(80–103)	▴0.256	98(96–98)	96(95–98)	▴0.152	70.2 ± 4.58	70.1 ± 4.39	^●^0.960
**T**₁	93 ± 9.2	97 ± 13.5	^●^0.181	106(94.8–114)	103(94–113)	▴0.581	99(98–100)	99(98–100)	▴0.588	76.4 ± 4.62	77.2 ± 5.48	^●^0.520
**T**₂	84 ± 9.9	89 ± 14.1	^●^0.090	101(55–127)	91(57–140)	▴0.415	99(98–100)	99(97–100)	▴0.719	74.1 ± 5.48	74.7 ± 5.27	^●^0.629
**T**₃	80 ± 9.2	83 ± 14.9	^●^0.224	88(68–118)	85(68–119)	▴0.474	98(97–100)	98(96–100)	▴0.650	73.8 ± 4.63	72.9 ± 4.90	^●^0.416
**T**₄	78 ± 12.1	80 ± 14.5	^●^0.558	83.5(68–109)	80(65–95)	▴0.235	98(96–99)	98(95–100)	▴0.959	72.9 ± 4.90	71.6 ± 4.52	^●^0.273
**T**₅	77 ± 11.2	79 ± 14.7	^●^0.518	80(76.3–84)	79(75–84)	▴0.176	98(96–99)	98(96–99)	▴0.562	72.1 ± 5.10	70.7 ± 4.92	^●^0.260
**T**₆	88 ± 12.1	86 ± 14.6	^●^0.597	91.5(84–103)	86(68–115)	▴0.081	96(96–99)	97(96–98)	▴0.598	72.6 ± 5.23	70.8 ± 4.21	^●^0.133
	**E: Δ HR(bpm)**			**F: Δ MAP(mmHg)**			**G: ΔSpO**_**2(**_**%)**			**H: ΔrSO**_**2(**_**%)**		
	**Group 12**	**Group 16**		**Group 12**	**Group 16**		**Group 12**	**Group 16**		**Group 12**	**Group 16**	
	**Mean ± SD**	**Mean ± SD**	***p* value**	**Median** **(Q1-Q3)**	**Median** **(Q1-Q3)**		**Median** **(Q1-Q3)**	**Median** **(Q1-Q3)**	***p* value**	**Mean ± SD**	**Mean ± SD**	***p* value**
**T₀-T**₁	+ 9.97	+ 13.52	▴0.369	+ 9.79	+ 11.91	▴0.915	+ 1.79	+ 3	▴0.365	+ 6.21	+ 7.06	▴0.446
**T₀-T**₆	+ 4.62	+ 2.61	▴0.455	-1.59	-3.15	▴0.522	+ 0.03	+ 1.55	▴0.053	+ 2.44	+ 0.73	▴0.172
**T**₁**-T**₂	-9.38	-8.09	▴0.980	-11.29	-9.52	▴0.900	-0.44	-0.48	▴0.517	-2.32	-2.48	▴0.659
**T**₂**-T**₃	-4.12	-5.52	▴0.237	-6.53	-8.70	▴0.221	-0.32	-0.33	▴0.692	-0.24	-1.82	▴0.029*
**T**₂**-T**₄	-5.47	-8.67	▴0.072	-10	-12.94	▴0.555	-0.59	-0.42	▴0.603	-1.15	-3.06	▴0.076
**T**₂**-T**₅	-7.15	-10.18	▴0.171	-12.03	-14.64	▴0.905	-0.50	-0.73	▴0.683	-1.97	-3.97	▴0.077

Sub-sections A–D present absolute physiological values, while Sub-sections E–H represent relative changes (Δ) between specific operative time points

**• A–D:** HR, heart rate (bpm); MAP, mean arterial pressure (mmHg); SpO_2_, peripheral oxygen saturation (%); rSO_2_, regional cerebral oxygenation (%)

**• E–H:** ΔHR, ΔMAP, ΔSpO_2_, and ΔrSO_2_ denote the calculated differences between the specified time intervals

**Time Points and Definitions:** T_0_: Baseline (supine, spontaneous breathing); T_1_: Post-intubation (supine, mechanical ventilation); T_2_: pneumoperitoneum (12 or 16 mmHg, supine); T_3_: pneumoperitoneum + reverse Trendelenburg (RT); T_4_: pneumoperitoneum + RT (30 min); T_5_: pneumoperitoneum + RT (60 min); T_6_: Desufflation (RT position)

Group 12 (12 mmHg pneumoperitoneum); Group 16 (16 mmHg pneumoperitoneum). ● Student's T-Test; ▴ Mann–Whitney U test. Data are presented as mean ± SD or median (Q1–Q3). Statistically significant differences (*p* < 0.05) are indicated with an asterisk (*) and shown in bold

**Fig. 2:**
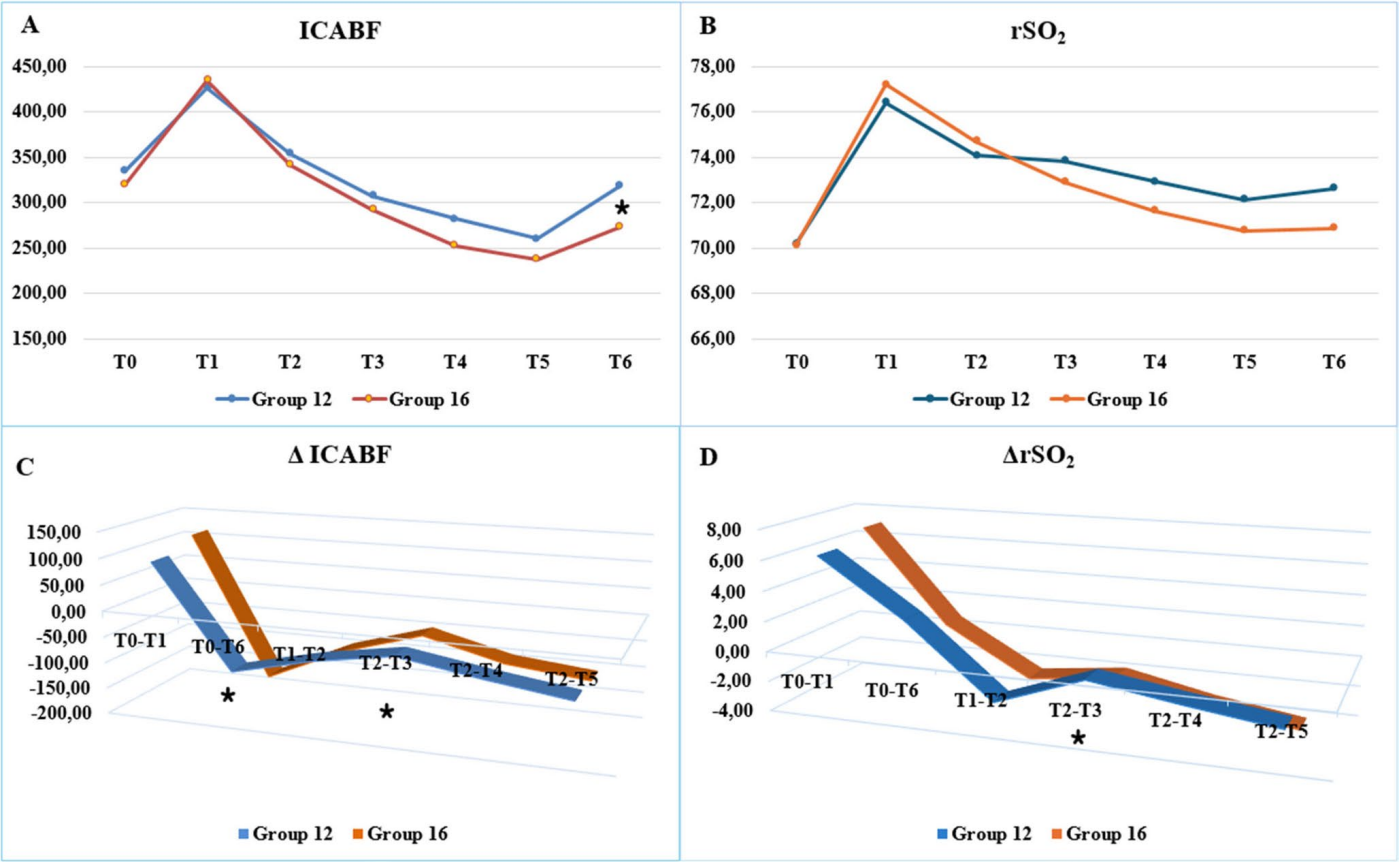
Longitudinal analysis of internal carotid artery blood flow (ICABF) and regional cerebral oxygenation (rSO_2_) across operative time points. This figure illustrates the relationship between cerebral perfusion and oxygenation levels in patients with severe obesity across two different intra-abdominal pressure settings. **• Panels A and B**: Absolute trends for ICABF (mL/min) and rSO_2_ (%) from baseline (T_0_) to post-desufflation (T_6_). **• Panels C and D**: Relative changes (Δ) for ICABF (mL/min) and rSO_2_ (%) calculated between specific operative time intervals. **Time Points and Definitions**: T_0_: Baseline (supine, spontaneous breathing); T_1_: Post-intubation (supine, mechanical ventilation); T_2_: pneumoperitoneum (12 or 16 mmHg, supine); T_3_: pneumoperitoneum + reverse Trendelenburg (RT); T_4_: pneumoperitoneum + RT (30 min); T_5_: pneumoperitoneum + RT (60 min); T_6_: Desufflation (RT position). Group 12 (12 mmHg pneumoperitoneum); Group 16 (16 mmHg pneumoperitoneum). Intergroup comparisons at each time point were conducted using the Student’s t-test or Mann-Whitney U test, depending on data normality. Bold values with an asterisk (*) indicate statistical significance (*p* < 0.05)

Absolute rSO_2_ values were similar between the groups (*P* > 0.05, Fig. 2B). However, delta scores during the transition from pneumoperitoneum to RT (T_2_–T_3_) revealed a significantly more pronounced rSO_2_ decrease in Group 16 than in Group 12 (Table 2 and Fig. 2D, *P* = 0.029). Baseline (T_0_) and induction (T_1_, T_2_) VTI values were also similar. However, Group 16 exhibited significantly lower VTI than Group 12 from T_3_ through T_6_ (Table 3, *P* < 0.05). While internal carotid diameters were similar (*P* > 0.05, Table 3), Group 16 exhibited a significantly lower ICABF at the final measurement point (T6) compared to Group 12 (Table 3 and Fig. 2A, *P* = 0.046).

**Table 3:** Intraoperative carotid artery hemodynamics: Absolute Doppler measurements and relative changes across time points

** VTI(cm)**				** ICAD(cm) **			** ICABF(mL/min)**		
	**Group12**	**Group16**		**Group 12**	**Group 16**		**Group 12**	**Group 16**	
	**Mean ± SD**	**Mean ± SD**	***p* value**	**Mean ± SD**	**Mean ± SD**	***p* value**	**Mean ± SD**	**Mean ± SD**	***p* value**
**T₀**	11.5 ± 1.64	11.1 ± 1.58	^●^ 0.254	0.66 ± 0.06	0.66 ± 0.06	^●^0.939	335.01 ± 87.5	319.26 ± 93.9	^●^ 0.480
**T**₁	12.1 ± 1.56	11.7 ± 1.72	^●^ 0.292	0.69 ± 0.06	0.70 ± 0.06	^●^0.780	425.49 ± 87.5	435.75 ± 118	^●^ 0.716
**T**₂	11.7 ± 1.66	11 ± 1.69	^●^ 0.065	0.67 ± 0.06	0.66 ± 0.07	^●^0.724	354.09 ± 105	340.58 ± 100	^●^ 0.593
**T**₃	11.4 ± 1.65	10.6 ± 1.63	^●^ **0.046**^**#**^	0.65 ± 0.06	0.64 ± 0.06	^●^0.791	307.62 ± 89.2	292.22 ± 92.1	^●^ 0.489
**T**₄	11.2 ± 1.62	10.2 ± 1.54	^●^ **0.020**^**#**^	0.64 ± 0.05	0.62 ± 0.06	^●^0.421	282.03 ± 82.5	252.64 ± 75.7	^●^ 0.134
**T**₅	11 ± 1.59	9.94 ± 1.60	^●^ **0.007**^**#**^	0.62 ± 0.06	0.62 ± 0.07	^●^0.955	260.22 ± 79.3	236.61 ± 71.9	^●^ 0.207
**T**₆	11.2 ± 1.63	10.1 ± 1.55	^●^ **0.006**^**#**^	0.64 ± 0.05	0.63 ± 0.06	^●^0.589	318.08 ± 94.8	272.86 ± 86.4	^●^ **0.046**^**#**^
** Δ VTI(cm)**				** Δ ICAD(cm)**			** Δ ICABF(mL/min)**		
	**Group12**	**Group16**		**Group 12**	**Group 16**		**Group 12**	**Group 16**	
	**Mean ± SD**	**Mean ± SD**	***p* value**	**Mean ± SD**	**Mean ± SD**	***p* value**	**Mean ± SD**	**Mean ± SD**	***p* value**
**T₀-T**₁	+ 0.61	+ 0.64	▴0.975	+ 0.03	+ 0.03	▴0.103	+ 90.48	+ 115.49	▴0.074
**T₀-T**₆	-0.33	-0.98	▴ < 0.001*	-0.03	-0.03	▴0.128	-107.41	-161.89	▴0.004*
**T**₁**-T**₂	-0.37	-0.72	▴ < 0.001*	-0.02	-0.03	▴0.137	-71.40	-94.17	▴0.171
**T**₂**-T**₃	-0.31	-0.35	▴0.408	-0.02	-0.02	▴0.939	-46.47	-48.36	▴0.443
**T**₂**-T**₄	-0.58	-0.74	▴0.151	-0.03	-0.04	▴0.207	-72.06	-87.95	▴0.036*
**T**₂**-T**₅	-0.72	-1.04	▴0.015*	-0.05	-0.04	▴0.791	-93.87	-103.98	▴0.510

*VTI*, velocity time integral (cm); *ICAD*, internal carotid artery diameter (cm); *ICABF*, internal carotid artery blood flow (mL/min); *Δ*, relative change between specified time intervals; Group 12 (12 mmHg pneumoperitoneum); Group 16 (16 mmHg pneumoperitoneum)

**Time Points and Definitions:** T_0_: Baseline (supine, spontaneous breathing); T_1_: Post-intubation (supine, mechanical ventilation); T_2_: pneumoperitoneum (12 or 16 mmHg, supine); T_3_: pneumoperitoneum + reverse Trendelenburg (RT); T_4_: pneumoperitoneum + RT (30 min); T_5_: pneumoperitoneum + RT (60 min); T_6_: Desufflation (RT position)

● Student’s t-test; ▴ Mann–Whitney U test. Bold values with an asterisk (*) indicate statistical significance (*p* < 0.05)

Group 16 demonstrated more pronounced VTI declines during the T_0_–T_6_ (*P* < 0.001), T_1_–T_2_ (*P* < 0.001), and T_2_–T_5_ (*P* = 0.015) intervals (Table 3). Similarly, ICABF reduction was significantly greater in this group during both T_0_–T_6_ (*P* = 0.004, Fig. 2C) and T_2_–T_4_ (*P* = 0.036) periods (Table 3).

## Discussion

This research evaluated how two distinct pneumoperitoneum pressure levels affect internal carotid artery hemodynamics and cerebral oxygenation in patients with severe obesity undergoing LSG. Our findings demonstrate that while systemic hemodynamic stability is preserved, an IAP of 16 mmHg significantly reduces ICABF and rSO₂ at a subclinical level compared to the 12 mmHg group. Notably, an elevated IAP of 16 mmHg imposes a subclinical hemodynamic burden by straining cerebral reserves through cumulative ICABF reductions and acute rSO₂ declines. This pattern, characterized by an immediate drop in oxygenation during the (T_2_–T_3_) interval and a persistent decline in arterial inflow that fails to recover even five minutes after desufflation (T_6_), suggests that higher pressure loads may strain the cerebral homeostatic buffering capacity in patients with severe obesity.

A primary observation of this study is the mismatch between routine systemic monitoring and regional cerebral perfusion dynamics. While standard markers—specifically MAP, HR, and SpO_2_—showed no statistical difference between the two pressure groups (*P* > 0.05) (Table 2), the pronounced decline in carotid artery parameters (VTI and ICABF) reflects a subclinical compromise in tissue perfusion. This corroborates the evidence from Yu et al. [6] suggesting that carotid flow monitoring detects perfusion changes before systemic hemodynamic alterations. By identifying this dissociation through internal carotid Doppler analysis, our findings highlight a potential influence of a 16 mmHg load on the compensatory margin of cerebral perfusion in patients with severe obesity, while remaining within subclinical limits throughout the procedure (T_0_–T_6_).

From a technical perspective, there is a noteworthy temporal divergence between the reductions in VTI and total ICABF. In Group 16, absolute VTI values decreased as early as the initial operative phase (T_3_), marking the point where the combined mechanical stress of pneumoperitoneum and RT position became most prominent. This synergy is known to elevate intrathoracic pressure and impede venous return, potentially challenging cerebral homeostatic mechanisms [3, 4]. Importantly, Δ comparisons for VTI across the total operative duration (T_0_–T_6_) revealed a significant and sustained reduction (Table 3), reflecting a cumulative compromise in stroke-by-stroke carotid inflow. While absolute ICABF values reached statistical significance only at the final stage (T_6_) (Table 3; Fig. 2A), Δ comparisons confirmed that the perfusion restriction was progressive, showing significant differences across the (T_1_–T_2)_ and (T_2_–T_5_) intervals (Table 3). The fact that both VTI and ICABF remained suppressed at (T_6_), five minutes after desufflation, while maintaining the RT position, indicates a delayed recovery pattern. Because ICA diameters remained constant (*P* > 0.05), this prolonged reduction in total flow is likely secondary to decreased flow velocity rather than caliber variations. These findings align with the principles described by Willie et al. [7], suggesting that cerebral vessels maintain stable diameters to regulate perfusion through modulations in flow velocity. This interpretation is further supported by the widely accepted methodological assumption in transcranial Doppler–based cerebrovascular studies that large cerebral artery diameters do not change substantially during short-term autoregulatory responses, allowing changes in flow velocity to serve as a surrogate for cerebral perfusion [8]. The timing discrepancy reflects the initial efficacy of cerebral autoregulation; however, this shift suggests that in patients with severe obesity, higher pressure loads may strain cerebral reserves, leading to a prolonged but subclinical reduction in perfusion capacity. Significant differences in delta comparisons, specifically for the (T_2_–T_4_) and (T_0_–T_6_) intervals, further support the deepening nature of this restriction in this cohort (Fig. 2C).

Cerebral oximetry findings aligned with the macrocirculatory trends identified through Doppler ultrasonography. Although absolute rSO_2_ levels were similar between the groups (Fig. 2B), the Δ values provided a more nuanced insight into regional oxygenation changes. Specifically, during the interval where the combined effects of pneumoperitoneum and RT positioning were most prominent (T_2_–T_3_), the Δ in oxygenation for Group 16 was greater than that of Group 12 (*P* = 0.029) (Table 2; Fig. 2D). In contrast to the cumulative and persistent nature of the ICABF reduction, the rSO_2_ decline appeared more acute, likely reflecting an immediate response to increased intrathoracic pressure and impaired venous return. These findings suggest that a 16 mmHg load may influence cerebral compensatory mechanisms in patients with severe obesity, particularly when additional positional stress is introduced [9]. These findings highlight how increased IAP affects the equilibrium of oxygen delivery and consumption [10].

Current evidence suggests that rSO_2_ delta values detect early perfusion deficits more effectively than absolute measurements [11, 12]. In this study, no patient fell below the 20% clinical intervention threshold. Such stability may be attributed to the strong vascular reserves typically found in younger cohorts [13]. However, statistical Δ analysis indicates a more substantial reserve consumption in Group 16 compared to Group 12. Although patients remained within safe limits, these findings suggest the 16 mmHg load is not physiologically inert. Such a load could imply that cerebral tissue may approach its compensatory limit during positional changes. While standard absolute monitoring might offer a potentially incomplete picture of clinical status, Δ analysis better reflects the subclinical reduction in perfusion reserve observed in patients with severe obesity.

Patients with severe obesity and concurrent cerebrovascular disease or carotid stenosis are particularly susceptible to high-pressure pneumoperitoneum. In this cohort, a 16 mmHg load may rapidly exhaust cerebral compensatory mechanisms [14]. This transition carries the potential to shift a subclinical state toward a clinical perfusion deficit. Furthermore, while long-term neurological outcomes were not monitored, the cumulative perfusion deficits observed in Group 16 underscore the importance of maintaining cerebral reserves in patients with severe obesity during prolonged procedures.

The primary mechanism for this hemodynamic impairment is the increase in IAP, which in turn elevates intrathoracic pressure. This elevation hinders cerebral venous drainage and increases cerebral venous pressure [15, 16]. The delayed recovery of ICABF observed at (T_6_) suggests that in patients with severe obesity, cerebral venous congestion or increased intracranial pressure may not resolve immediately upon desufflation. Akkurt et al. [17] noted that pressure levels during LSG directly affect the diameter and drainage capacity of the internal jugular vein. Reduced chest wall compliance and constrained physiological reserves may promote persistent cerebral venous congestion in patients with severe obesity [18, 19]. Additionally, evidence from recent meta-analyses and international consensus statements on enhanced recovery after surgery (ERAS) supports the notion that low-pressure pneumoperitoneum is associated with a stable hemodynamic profile while maintaining surgical safety [20–22]. This approach aligns with contemporary perioperative care standards designed to mitigate physiological stress and preserve patient reserves during laparoscopic procedures.

### Limitations

Certain limitations should be acknowledged. Primarily, cerebral perfusion was evaluated using ICA flow dynamics and NIRS rather than transcranial Doppler. While ICA flow may not reflect the full complexity of intracranial microcirculation, it serves as a primary source of cerebral inflow. The alignment of these measurements with our NIRS data suggests a reasonable assessment of general perfusion. Furthermore, while this study focused on arterial inflow and regional oxygenation, cerebral perfusion is also dependent on the balance of venous outflow. The lack of jugular venous Doppler assessment, which could be influenced by increased intrathoracic pressure during pneumoperitoneum, remains a limitation of our work. Future studies incorporating venous drainage dynamics would provide a more comprehensive understanding of cerebral homeostatic buffering in this patient population.

Additionally, our study did not investigate intermediate pressure levels such as 14 or 15 mmHg; these values remain a potential subject for further evaluation. Our observations were also limited to the intraoperative period, meaning the long term neurocognitive effects of the detected subclinical shifts were not evaluated. Furthermore, although we ensured deep clinical paralysis through a standardized rocuronium protocol, the lack of objective neuromuscular monitoring remains a limitation, as it prevented the precise quantification of the depth of paralysis. Future research incorporating objective monitoring could further refine the relationship between muscle relaxation and intra-abdominal pressure readings. Prospective studies are needed to explore whether these hemodynamic fluctuations have clinical relevance beyond the immediate perioperative period.

Finally, a study design comparing different groups was preferred over a crossover design where patients served as their own controls to isolate the cumulative and progressive physiological burden of a constant pressure level throughout the procedure. Given that each measurement required a stabilization period of 5 min to ensure a physiological steady state, alternating between different pressures within the same patient would have introduced significant carryover effects. This methodological choice was made to avoid confounding the assessment of time-dependent hemodynamic changes and to prevent an unnecessary prolongation of the surgical duration.

## Conclusion

Our data demonstrate that, relative to 12 mmHg, a 16 mmHg pneumoperitoneum induces a gradual reduction in ICABF and abrupt declines in rSO_2_ in patients with severe obesity. Notably, these subclinical alterations were not directly reflected in systemic parameters such as MAP and SpO_2_, which remained within stable limits throughout the procedure. These findings suggest that higher insufflation pressures may subclinically strain cerebral compensatory reserves without immediate warning. Therefore, an individualized approach centered on the minimum pressure at which adequate surgical vision is maintained merits clinical attention. This strategy may offer a safer margin for preserving cerebral perfusion in patients with severe obesity. Ultimately, this approach aligns with contemporary perioperative care standards designed to mitigate physiological stress and preserve patient reserves during laparoscopic procedures.

## Data Availability

The data that support the findings of this study are available from the corresponding author upon reasonable request.
